# Exercise training and DNA methylation profile in post-bariatric women: Results from an exploratory study

**DOI:** 10.3389/fspor.2023.1092050

**Published:** 2023-02-08

**Authors:** Carolina F. Nicoletti, Hamilton Roschel, Carlos Merege-Filho, Alisson P. Lima, Saulo Gil, Marcela A. S. Pinhel, Natalia Y. Noronha, Marco A. Santo, Amalia Jacome, Ana B. Crujeiras, Bruno Gualano, Carla B. Nonino

**Affiliations:** ^1^ Department of Internal Medicine, Ribeirão Preto Medical School, University of Sao Paulo, Ribeirão preto, Brazil; ^2^ Applied Physiology & Nutrition Research Group, School of Physical Educaton and Sport, Rheumatology Division, Faculdade de Medicina FMUSP, Universidade de Sao Paulo, Sao paulo, Brazil; ^3^ Laboratory of Studies in Biochemistry and Molecular Biology, Department of Molecular Biology, São José do Rio Preto Medical School, Sao Jose do Rio Preto, Brazil; ^4^ Department of Digestive Surgery, School of Medicine, University of Paulo, Sao Paulo, Brazil; ^5^ Department of Mathematics, MODES Group, CITIC, Universidade da Coruña, Faculty of Science, A coruña, Spain; ^6^ Epigenomics in Endocrinology and Nutrition, Instituto de Investigación Sanitaria (IDIS), Complejo Hospitalario Universitario de Santiago (CHUS) and Santiago de Compostela University (USC), Santiago de Compostela, Spain, CIBER Fisiopatología de la Obesidad y la Nutrición (CIBERobn), Madrid, Spain; ^7^ Department of Health Science, Ribeirão Preto Medical School, University of Sao Paulo, Ribeirão preto, Brazil

**Keywords:** obesity, DNA methylation, epigenetic, exercise program, th7 cell differentiation

## Abstract

Exercise training and bariatric surgery have been shown to independently modulate DNA methylation profile in clusters of genes related to metabolic and inflammatory pathways. This study aimed to investigate the effects of a 6-month exercise training program on DNA methylation profile in women who underwent bariatric surgery. In this exploratory, quasi-experimental study, we analyzed DNA methylation levels by array technology in eleven women who underwent Roux-en-Y Gastric Bypass and a 6-month, three-times-a-week, supervised exercise training program. Epigenome Wide Association Analysis showed 722 CpG sites with different methylation level equal to or greater than 5% (*P* < 0.01) after exercise training. Some of these CpGs sites were related to pathophysiological mechanisms of inflammation, specially Th17 cell differentiation (FDR value < 0.05 and *P* < 0.001). Our data showed epigenetic modification in specific CpG sites related to Th17 cell differentiation pathway in post-bariatric women following a 6-months exercise training program.

## Introduction

Epigenetics mechanisms underlying obesity and weight loss therapy have received a lot of attention in clinical research (1). In this context, DNA methylation has been widely studied as an epigenetic marker due to its influence in gene expression (2). Different strategies to control obesity and promote weight loss, such as hypocaloric dietary intervention (3), bariatric surgery (4) and exercise training program (5) have been studied and associated with changes in DNA methylation profile. For instance, we previously identified wide changes in CpG sites related to metabolism, immunity, and inflammation following bariatric surgery (6). In fact, DNA methylation level of 666 CpGs sites were hypermethylated after bariatric procedure (4).

It is noteworthy that some cluster of genes associated with obesity appear unchanged following surgical procedure (4), warranting the search for others therapies capable of boosting the benefits of bariatric surgery. In this regard, exercise training emerges as a potential adjuvant therapy able to modulate DNA methylation of distinct genes related to adipocyte metabolism (7) and inflammatory responses (8), with were related to reduced risk of chronic diseases (9). Study evaluating the effects of three exercise modalities on DNA methylation levels observed that the practice of resistance training increased global DNA methylation (8).

Despite data suggested that obesity is related to a peculiar DNA methylation patterns which may be mitigated after weight loss therapy (3, 10), the evidence is still limited, even more when two strategies are carried out together. Thus, we investigated the effects of a 6-month exercise training program on DNA methylation profile in women who underwent bariatric surgery.

## Material and methods

This exploratory, quasi-experimental study is part of a large randomized controlled trial (clinicaltrials.gov: NCT02441361), which is thoroughly describe elsewhere (11, 12). This study has been approved by the Clinical Hospital of the School of Medicine of the University of Sao Paulo in Brazil (HCFMUSP)´s Ethical Committee. All participants gave their written consent for participation in the study.

### Participants

Eleven women who underwent Roux-en-Y Gastric Bypass were consecutively enrolled from a Bariatric and Metabolic Surgery Unit in a Brazilian public hospital. Women with body mass index (BMI) > 40 kg/m^2^ or ≥35 kg/m^2^ with associated co-morbidities, aged between 18 and 55 years, and not engaged in an exercise training program for at least one year prior to the study were considered eligible. Exclusion criteria involved cancer in the past 5 years, and any cardiovascular diseases, neurological disorders or skeletal muscle impairment that would contraindicate exercise practice. A detailed description of the study protocol has been published previously (11, 12).

Three months after surgery, all participants participated in a 6-months, three-times-a-week, supervised exercise training program at the hospital. Weight, height, BMI, abdominal circumference and body composition were evaluated before and after the exercise training program. Also, before and after intervention, blood sample were collected after 12 h fasting and storage at −80 °C until epigenetic analysis.

### Exercise training program

Training sessions included a 5 min light warm-up followed by strengthening exercises for the major muscle groups (leg-press 45^o^, leg extension, half-squat, bench press, let pulldown, seated row and calf raise) and aerobic exercise on a treadmill. The resistance exercise protocol was comprised of three sets of 8–12 repetition maximum with a 60-second rest interval between sets and exercises. Load progression (5%) was employed as soon as participants were able to perform two or more repetitions than previously determined. Aerobic training consisted of 30–60 min (10 min progression every 4 weeks) of treadmill walking at an intensity corresponding to 50% of the delta difference between the ventilatory anaerobic threshold and respiratory compensation point. Heart rate was monitored throughout every session to ensure proper exercise intensity (Polar®).

### DNA methylation analysis

Genomic DNA were extracted from peripheral blood and then was bisulphite converted and hybridized to Illumina Infinium Methylation EPIC Beadchip Microarray (EPIC-array, San Diego, CA, United States) according to the manufacturer's protocols. Beadchips were scanned with the Illumina iScan System (RRID:SCR_016388) and image intensities were extracted with the Genome Studio (2011.1) Methylation Module (v1.8.5). All filters and internal controls were performed as described before (4, 13). The probes that were considered single nucleotide polymorphisms were filtered out. To quantify methylation levels of each CpG sites, raw data were converted into *β*-values using the Genome Studio software (Illumina, San Diego, CA, United States). The methylation level was expressed as a beta (*β*) value that was calculated as the intensity of the methylated channel divided by the total intensity (*β* = Max (SignalB, 0)/(Max (SignalA, 0) + Max (SignalB, 0) + 100)). *β* values range from 0 (unmethylated) to 1 (fully methylated) and was interpreted as the percentage of CpG methylation. For genomic regions, DNA methylation was calculated as the mean *β* for all the probes located within the region annotated by Illumina: TSS200 (TSS - transcription start site), TSS1500, 5′UTR (UTR -untranslated region), 1st Exon, gene body, 3′UTR, and intergenic.

### Statistical analysis

Clinical characteristics are presented as mean ± standard deviation. Shapiro-Wilk test was performed to verify data normality. Paired t-test was carried out to compare the timepoints (before exercise training vs. after exercise training). All analyses were conducted with the Statistical Package software for Social Sciences (SPSS version 20.0, Inc. Chicago, IL) and significance level was set at *P* < 0.05.

Epigenome Wide Association Analysis (EWAS) was performed using the differential methylation analyses aimed to find differences in DNA methylation levels within the same subject over time points (before and after exercise training program). For this, parametric t-test and R statistical software (version 3.5.1) were used. EWAS were adjusted for multiple testing using Bonferroni correction, in which a false discovery rate (FDR) < 0.05 was considered statistically significant. However, given the limited sample size, raw *P* values of 0.01 were selected as a less stringent cutoff for differential methylation than q values. Also, a threshold for the significant CpG sites based on *Δβ* with a minimum value of 5% was applied.

Finally, to find functional interpretation of the results using the significant findings from EWAS as input, enriched KEGG (Kyoto Encyclopedia of Genes and Genomes) pathways were analyzed in WEBGestalt (WEB-based GEne SeT AnaLysis Toolkit) database. The human genome was used as a reference, and the enrichment *P* values were derived from a modified Fisher's exact test.

## Results

We observed a significant decrease in BMI and fat mass after 6-months exercise training (Table 1). EWAS analysis evidenced 722 CpG sites (related to 433 unique genes) that exhibited differential methylation equal to or greater than 5% (*P* < 0.01). The significant difference varied from 10 to 42%.

**Table 1:** Clinical characteristics of women who underwent bariatric surgery before and after a six months of exercise training protocol.

	Before exercise training	After exercise training	*p* value
Weight (kg)	92.6 ± 11.4	77.7 ± 9.9	0.013
Height (m)	1.6 ± 0.1	–	–
BMI (kg/m^2^)	35.7 ± 3.3	30 ± 2.5	0.004
Fat mass (%)	48.4 ± 3.9	37.7 ± 6.2	0.001
Fat free mass (%)	51.6 ± 4.1	62.3 ± 6	0.001
Total cholesterol (mg/dl)	168 ± 41,6	160,7 ± 23,9	0.988
LDL cholesterol (mg/dl)	100,3 ± 30	86,3 ± 19,8	0.057
HDL cholesterol (mg/dl)	44,9 ± 9,6	56,1 ± 13,8	0.033
Triglycerides (mg/dl)	98,5 ± 30,1	91 ± 26	0.566
Glycemia (mg/dl)	82,1 ± 14	77,8 ± 8,2	0.957

Data present average ± standard deviation. Paired t test. BMI: body mass index; LDL: low density lipoprotein; HDL: high density lipoprotein.

**Figure 1:**
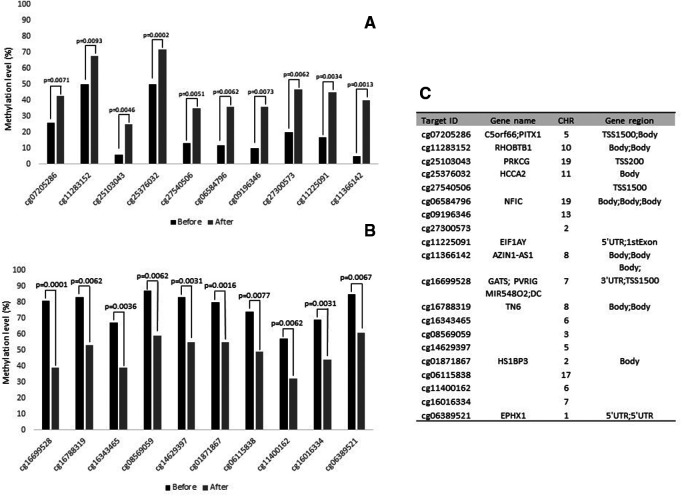
Top 20 CpG sites that were differently methylated after physical intervention in bariatric surgery women. (**A**): Hypermethylated CpGs sites after intervention. (**B**): Hypomethylated CpGs sites after intervention. (**C**): Characteristics of CpG sites. CHR: chromosome.

**Figure 2:**
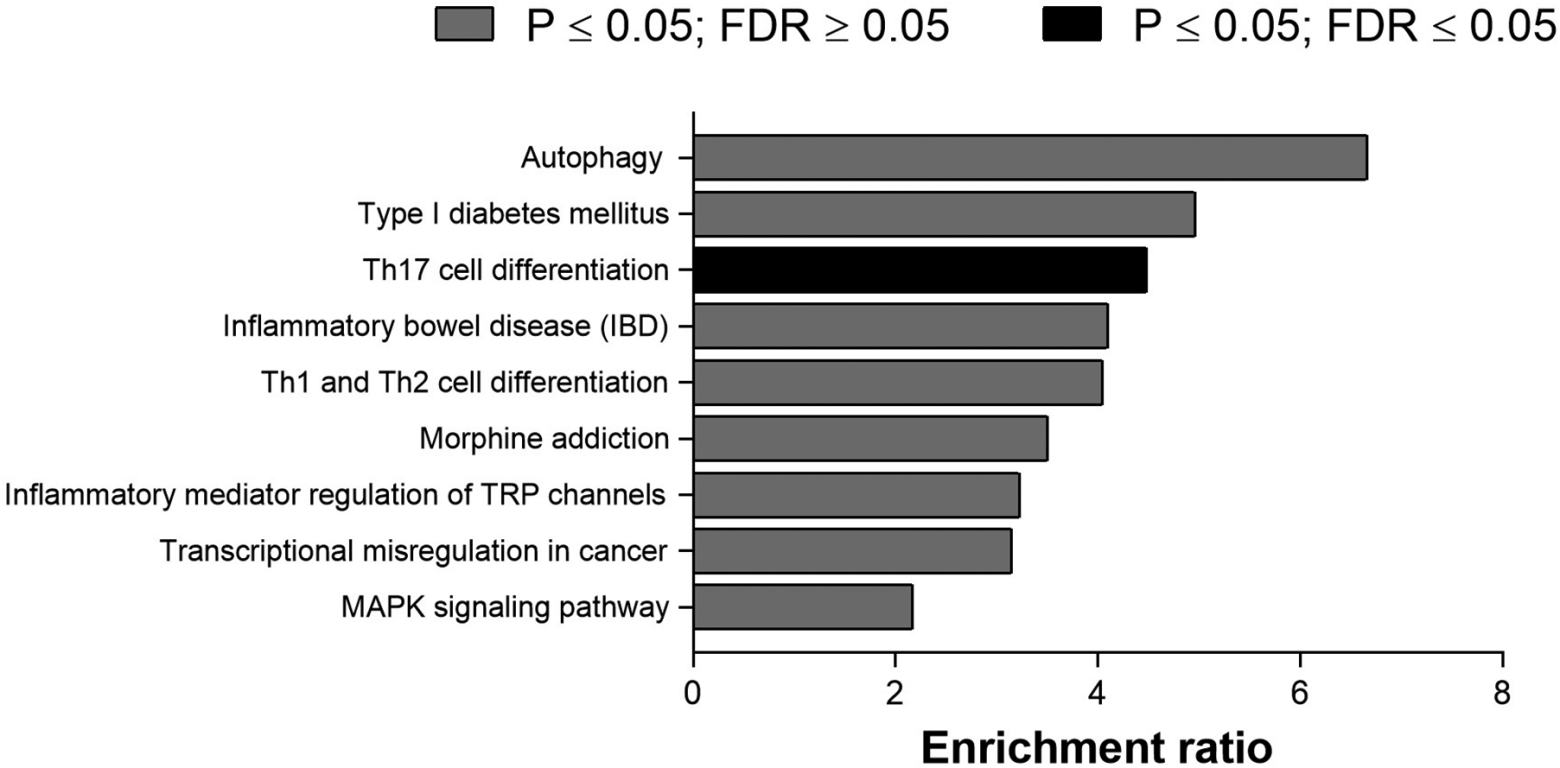
Top 10 canonical pathways of differentially methylated CpG sites after physical intervention in bariatric surgery women.

Figure 1 shows the top 20 differently methylated CpG sites before and after exercise training. The most significant differences were observed for cg11366142 and cg16699528 in the AZIN1-AS1 and GATS genes, respectively. Regarding metabolic pathways, the top 10 canonical pathways selected were described in Figure 2. Only Th17 cell differentiation pathway shows FDR value < 0.05 (and *P* < 0.001).

The specific changes in CpGs sites according to Th17 pathwaýs genes are descried in Table 2. There was an increase in methylation levels of CpG sites related to HLA-DOA, IL1RAP and NFATC3 genes, and a decrease in methylation levels of CpG sites located at HLA-DMB, HLA-DQB1, IL23R, IL2RA and MAPK10 genes.

**Table 2:** Cpgs sites in genes related to Th17 cell differentiation pathway changed with exercise training.

Gene symbol	Decrease methylation level	Increase methylation level
HLA-DMB	cg24523259	–
HLA-DOA	–	cg1563095
HLA-DQB1	cg10989981	–
IL1RAP	–	cg18230049
IL23R	cg120560449	–
IL2RA	cg17281724	–
MAPK10	cg10682568	–
NFATC3	–	cg27095776
TGFBR2	cg06850459	–

## Discussion

EWAS study in whole blood of post-bariatric women revealed 722 CpG sites with DNA methylation levels changed after exercise training. Some of these CpGs sites have been suggested to be involved in the pathophysiological mechanisms of inflammation, specially the Th17 cell differentiation, which is recognized to be a subset of pro-inflammatory T helper cells defined by their production of interleukin- 17. To our knowledge, this is the first EWAS investigating the whole blood methylation profile after longitudinal exercise training program in post-bariatric surgery participants.

Exercise is known to promote a strong anti-inflammatory response, independently of weight loss (14), which seems to occur by reducing the amount of pro-inflammatory cytokine and/or increasing anti-inflammatory cytokines (15). A recent review evidenced that exercise induced anti-inflammatory effects which may be due to epigenetic changes (16). Our findings indicated a prominent DNA methylation changes in Th17 cell differentiation pathway following exercise training. These results corroborate with previously literature. Machado et al., 2021 evidenced that the methylation levels in promoter regions of IL-17A gene was higher in participants that were submitted to exercise program when compared to the control group (8). According to the authors, the exercise-induced increase of DNA methylation in inflammatory genes could be related to loss of immune function (8).

Th17 cells characterize a subclass of CD4+ T cells that can produce interleukin-17 (IL-17) (17), which plays a role on body weight control, adipocyte differentiation, insulin and glucose homeostasis (18), and low-grade sustained inflammation related to obesity (19). Our findings suggest a modulatory effect of exercise in obesity-related chronic inflammation through changes in DNA methylation patterns in women with obesity who underwent bariatric surgery. In this sense, hypermethylation of Th17 pathway genes may reduce cytokine secretions capacity and decrease pro-inflammatory condition. Importantly, this condition might be related to improvement of metabolic state.

Despite the evidence that exercise induces changes in DNA methylation in the entire human genome, potentially affecting metabolism and inducing phenotypic adaptations that can result in improved general health, there is scant information about the optimal exercise dose/type able to elicit beneficial effects on epigenome associated with improvements in obesity management (20). Also, The main limitation of this study is the lack of controls (non-exercised and non-operated groups), which hampers any firm conclusions regarding the causative relationships between exercise and bariatric surgery and the observed changes in methylation patterns. Also, sample size was limited but the statistical significance shows a real difference between periods.

In conclusion, DNA methylation changes in specific CpG sites related to Th17 cell differentiation pathway was observed in post-bariatric women following a 6-months exercise training program. Data herein show the plasticity of the epigenomic associated with bariatric surgery and exercise training program, which advances our knowledge on the epigenomic effects of these combined interventions. These data should be confirmed by further randomized controlled studies. Considering the reversible nature of epigenetic modifications, their application in clinical practice is promising for future therapeutic strategies. The development of personalized epigenetics may drive a major expansion of accessible tools to clinicians in the management of obese patients submitted to bariatric surgery.

## Data Availability

The authors acknowledge that the data presented in this study must be deposited and made publicly available in an acceptable repository, prior to publication. Frontiers cannot accept a manuscript that does not adhere to our open data policies.
